# Synergistic Interaction Between Paired Combinations of Natural Antimicrobials Against Poultry-Borne Pathogens

**DOI:** 10.3389/fmicb.2022.811784

**Published:** 2022-05-04

**Authors:** Xiaoxia Liu, Rui Liu, Ruting Zhao, Jishi Wang, Yongyou Cheng, Qian Liu, Yanyun Wang, Shuming Yang

**Affiliations:** ^1^Key Laboratory of Agro-Product Quality and Safety, Institute of Quality Standards and Testing Technology for Agro-Products, Chinese Academy of Agricultural Sciences, Beijing, China; ^2^School of Investigation, People’s Public Security University of China, Beijing, China; ^3^Key Laboratory for Environmental Factors Control of Agro-Product Quality Safety, Ministry of Agriculture, Agro-Environmental Protection Institute, Ministry of Agriculture and Rural Affairs, Tianjin, China

**Keywords:** natural antimicrobials, antibiotic resistance, poultry-borne bacteria, minimum inhibitory concentrations, synergistic effect, combination index

## Abstract

Natural antimicrobials (NAM) are promising candidates for the successful control of poultry-borne bacteria, carrying potent antimicrobial activity (AMA) against a wide range of multidrug-resistant pathogens. Individual activities of carvacrol, eugenol, trans-cinnamaldehyde, oregano, and thymol, along with the combined activity of paired compounds, were examined using broth microdilution and checkerboard techniques. The characteristic interactions between the compounds were calculated using an improved method, based on combination index (CI) values. The bacteria examined herein were selected due to their known genetic resistance to at least one antibiotic. Our results indicated that thymol was most effective, exhibiting the lowest minimum inhibitory concentration (MIC) value against *Salmonella pullorum*, *Escherichia coli*, and *Klebsiella pneumoniae*, establishing the order of antimicrobial efficacy as: thymol > oregano > carvacrol > trans-cinnamaldehyde > eugenol. In the interaction study, the paired combination of carvacrol and thymol showed synergistic effects and was highly effective in reducing the antibiotic resistance of all the evaluated pathogens. Notably, all CI values were <1.0 in evaluations of *S. pullorum*, indicating the absence of antagonism between eugenol and thymol (or oregano). In *K. pneumoniae*, majority of CI values, which had a few concentration points, were smaller than 1.0, indicating a synergistic effect between eugenol and carvacrol (oregano or thymol), and trans-cinnamaldehyde and carvacrol. In *E. coli*, apart from some concentration points, some CI values were smaller than 1.0, demonstrating a synergistic effect between eugenol and carvacrol, and thymol and carvacrol (eugenol or oregano). It is therefore of great significance to investigate and illuminate the minimal effect concentration of these five components when they are used in combination as feed additives. Moreover, the improved evaluation method of this study provides a precise and extensive means to assess the synergistic effects of NAM.

## Introduction

The incidence of antimicrobial resistance (AMR) is increasing. Based on the World Health Organization, approximately 700,000 mortalities are attributed to drug-resistant infections each year. Without necessary action against these infections, the death rate can potentially rise to 10 million deaths annually by 2050, with AMR forcing approximately 24 million people into abject poverty by 2030 (Courtenay et al., 2019). The poultry industry is highly dependent on antibiotics in feed to manage pathogens (Van Boeckel et al., 2015); however, the increase in acquired bacterial antibiotic resistance is a concern to both the poultry industry and consumers. Public concern over the use of dietary antibiotics in consumable meat production, especially in relation to antibiotic resistance formation in bacteria, has elevated the demand for developing alternatives to antibiotics for use in farm animals. Therefore, there is a great interest in the development of natural antimicrobials (NAM), which can successfully manage multidrug-resistant pathogens and provide a worthwhile alternative to antibiotics (Wright, 2019; Yang et al., 2019; Drame et al., 2020; Naeim et al., 2020). Furthermore, this trend has emerged due to the European Union and China banning the use of antibiotics as growth promoters in livestock or other agricultural domains. The so-called antimicrobial growth promoters (AGPs) and non-AGPs, which are non-antibiotic feed supplements that focus on increasing animal development and improving their health status, could be effectively used to control salmonellosis and other diseases caused by foodborne pathogens in animal sectors (Shan et al., 2007; Aljumaah et al., 2020).

Currently, NAM have excellent potential in successfully managing pathogens, particularly due to their potent antimicrobial activity (AMA) against a wide range of bacterial pathogens (Singh et al., 2015; Aziz and Karboune, 2018; Sima et al., 2018). NAM have garnered considerable attention owing to their non-toxic nature and rising concern over the safety of synthetic chemicals, which have historically been used as food preservatives and flavor enhancers (Salamci et al., 2007). Phenolic isomers, carvacrol, and thymol are the active ingredients in oregano oil (*Origanum glandulosum*). Trans-cinnamaldehyde is an aromatic aldehyde present in cinnamon bark extract (*Cinnamomum zeylandicum*), and eugenol is a polyphenolic compound found in clove oil (*Eugenia caryophillis*) (Jensen, 2002; Mattson et al., 2011). All these compounds are described as “generally recognized as safe” (GRAS) by the Food and Drug Administration (Lambert et al., 2001; Venkitanarayanan et al., 2013).

Although several studies have examined the activity of NAM against numerous discrete microbes, including pathogens borne in various ways (Friedman et al., 2002; Kollanoor Johny et al., 2010; Venkitanarayanan et al., 2013; Upadhyaya et al., 2016; Sima et al., 2018; Pinkerton et al., 2019), different studies have varying antimicrobial sample sources, testing methods and bacterial strains, indices to evaluate the results (Fyfe et al., 1998; Ettayebi, 2000; Delaquis, 2002). Thus, it is necessary to adopt an effective and standardized method to evaluate and quantify the synergistic roles of natural antimicrobial combinations.

Here, the discrete activities of carvacrol, eugenol, trans-cinnamaldehyde, oregano, and thymol, and their activities in paired combinations, were examined using the broth microdilution and checkerboard techniques. The checkerboard method (Shin and Kang, 2003; Filoche et al., 2005; Si et al., 2008) combined with combination index (CI) values was used to test the antimicrobial abilities of the compounds. The CI values are commonly used for the assessment of antimicrobial properties of NAM. In addition, the synergistic effects of the components individually or along with organic acids were tested against foodborne pathogens using the fractional inhibitory concentration (FIC) and EC indices (Franzot and Casadevall, 1997; Zhou et al., 2007a,b).

The five components have strong antibacterial effects, but their application in animals is not restricted by their flavor. Herein, the efficacies of NAM against antibiotic-resistant bacteria were assessed. The aim of this research was to compare the AMA of NAM and develop a standard parameter for comparing dose-response data. Moreover, the effective concentrations of paired combinations of carvacrol, eugenol, oregano, thymol, and trans-cinnamaldehyde against poultry-borne pathogens were reduced. We also evaluated the synergistic interaction of these agents against resistant target bacteria. Furthermore, an enhanced technique was established for the evaluation and quantification of the synergistic combinations. It is well-understood that the bacterial growth-inhibition mechanisms of many NAM provide a safe, efficient, and cost-effective way of managing antibiotic resistance in the food animal production chain.

## Materials and Methods

### Antimicrobial Agents

Selected NAM that are active against target bacterial pathogens were used in this study. Analytical grade trans-cinnamaldehyde (99% purity), eugenol (99% purity), carvacrol (98% purity), thymol (99% purity), geraniol (99% purity), linalool (99% purity), and citral (99% purity), as well as dimethyl sulfoxide (DMSO, 99.9%), were acquired from Sigma-Aldrich (St. Louis, MO, United States). Oregano (98% purity), mugwort (98% purity), and curcumin were obtained from RHAWN (Shanghai, China).

### Bacterial Strains and Inoculum Preparation

*Escherichia coli* 8G4 and *Klebsiella pneumoniae* 208G28, both strains derived from broiler chickens at the Institute of Quality Standards and Testing Technology for Agro-Products of the Chinese Academy of Agricultural Sciences CAAS (Beijing, China), were used for analysis. Three strains—*E. coli* BNCC 336435, *K. pneumoniae* BNCC 102997, and *Salmonella pullorum* BNCC 19945—were purchased from Jiangsu BeNa Culture Collection (Jiangsu, China) and used in this study. All bacteriological media were acquired from Beijing Land Bridge Technology Co., Ltd. (Beijing, China). Bacterial stock cultures were maintained at −80°C in nutrient broth (NB) medium (Beijing Land Bridge Technology Co., Ltd., Beijing, China) containing 15% glycerol. Working cultures of the poultry-borne pathogens were maintained at 4°C on nutrient agar (NA) medium (Beijing Land Bridge Technology Co., Ltd., Beijing, China). Prior to the experiment, a single colony was transferred from NA to 10.0 ml NB, followed by overnight incubation at 37°C with shaking at 150 rpm. Subsequently, 100 μl of bacterial suspension was added to 9.9 ml NB before incubation at 37°C for 12 h to acquire log phase cultures. Bacterial density was adjusted to 1 × 10^9^ CFU/ml using the Infinite M200 PRO microplate reader (TECAN, San José, CA, United States) and diluted to an optical density (OD) of 0.1 (approximately 1 × 10^6^ CFU/ml) at 600 nm.

### Minimum Inhibitory Concentrations Determination

Minimum inhibitory concentrations (MICs) of NAM indicating bacterial resistance were established using the broth microdilution assay, following a protocol described previously (Wiegand et al., 2008), with some alterations. Initially, we selected more than a dozen different types of NAM to inhibit poultry-borne pathogenic bacteria, after consulting the literature. Subsequently, five plant extracts (trans-cinnamaldehyde, eugenol, carvacrol, thymol, and oregano) were selected as study variables. A certain amount of each plant extract component was dissolved in DMSO and added to NB, to obtain a stock solution of approximately 2,000 μg/mL.

All natural antimicrobial solutions were prepared and filter-sterilized using 0.22 μm-pore filters (Millipore Sigma, Bedford, MA, United States) before each use. A minimum of eight concentrations in a geometric series and one control were prepared for each chemical. First, 200 μL of diluted antimicrobial solution was added to the Corning Costar^®^ 96-well microplate (Corning Incorporated, NY, United States). Thereafter, 20μL of the prepared bacterial solution was inoculated before plate incubation at 37°C for 24 h with shaking at 150 rpm. Finally, absorbance was detected using the Infinite M200 PRO microplate reader (TECAN) at 600 nm. A minimum of three distinct experiments were performed for each compound, and the results are presented as the percentage inhibition rate (%) in relation to control. The lowest concentration of NAM required to completely inhibit the growth of the tested microorganism was designated as the MIC.

### Screening Drug Combinations With Synergistic Effects Based on a Combination Index Model

Herein, we applied the CI method to assess the interactions of the compounds, derived from the median effect principle (Chou and Talalay, 1984). The CI-isobologram equation, which is based on major biochemical and biophysical equations from the median-effect equation, was employed for the assessment of the interactions of trans-cinnamaldehyde, eugenol, carvacrol, thymol, and oregano. For the combination of the compounds, the following equation was applied:


(1) $${\left(C\,I\right)}_{x}=\sum_{j=1}^{n}\frac{{\left(D\right)}_{j}}{{\left(D_{x}\right)}_{j}}=\sum_{i=1}^{n}\frac{{\left(D_{x}\right)}_{1-n}\,\left\{\frac{{\left[D\right]}_{j}}{\sum_{1}^{n}\left[D\right]}\right\}}{{\left(D_{m}\right)}_{j}\,{\left\{\frac{{\left(f_{a\,x}\right)}_{j}}{\left[1-{\left(f_{a\,x}\right)}_{j}\right]}\right\}}^{1/m\,j}}$$

where *^*n*^(CI)x* is the CI of *n* compounds at x% suppression, *(D_*x*_)1–n* is the sum concentrations of *n* compounds that produce x% synergistic suppression, *(D_*x*_)_1–n_{([D]_*j*/_*$\sum_{1}^{n}\left[D\right]\}$ is the proportional concentration of each *n* compound that produce x% synergistic suppression; and *(D_*m*_)j{f_*ax*_)j/[1–(f_*ax*_)j]^1/mj^* is the concentration of discrete compound that produces x% suppression, in which *D*_*m*_ is the median-effect dose, *f*_*ax*_ is the fractional suppression at x% suppression, and *m* is the slope of the median-effect plot. The Chou-Talalay technique is often employed in drug combination investigations and offers an estimation of the drug synergistic effect by computing the CI. Accordingly, CI < 1 indicates synergistic effect, CI < 0.5 indicates highly synergistic outcome, CI = 1 indicates additive outcome, and CI > 1 indicates antagonistic effect (Chou, 2006). The types of interactions between NAM were determined using CompuSyn software 2.0.

### Statistical Data Analysis

Half-maximal inhibitory concentration (IC50) and MIC values were obtained by fitting the dose-response curve of the antibiotics using advanced biometric software, such as SPSS and Origin. One-way analysis of variance (ANOVA) and Duncan’s multiple comparison test using the Origin software were used to analyze the experimental data and marked differences (*P* < 0.05) between means of both groups. The CompuSyn computer program was employed for the determination of the concentration-effect curve parameters and CI values.

## Results

### Minimum Inhibitory Concentrations of Natural Antimicrobials

Table 1 summarizes the MICs of 10 NAM producing distinct effects against poultry-borne pathogenic bacteria. Generally, NAM included in the evaluation were effective against the relevant pathogenic bacteria. Growth of *E. coli* was inhibited by carvacrol at 241.4 μg/mL, trans-cinnamaldehyde at 519.3 μg/mL, eugenol at 502.0 μg/mL, and oregano at 241.4 μg/mL. At 198.4 μg/mL, thymol was effective against *S. pullorum*, *E. coli*, and *K. pneumoniae*. Furthermore, carvacrol (167.4 μg/mL), trans-cinnamaldehyde (411.2 μg/mL), eugenol (502.0 μg/mL), thymol (198.4 μg/mL), and oregano (232.2 μg/mL) inhibited the growth of *S. pullorum*. Additionally, thymol concentration needed to inhibit the growth of all microorganisms was lower than that of the other NAMs.

**TABLE 1:** Minimum inhibitory concentrations (MIC) values of natural antimicrobials against Poultry-borne pathogens.

Antimicrobials	Bacteria (MIC μg/mL)				
	
	*Salmonella pullorum* 19945	*Escherichia coli* 336435	*Escherichia coli* 8G4	*Klebsiella pneumoniae* 102997	*Klebsiella pneumoniae* 208G28
Carvacrol	167.4	241.4	241.4	241.4	241.4
Trans-Cinnamaldehyde	411.2	519.3	519.3	411.2	519.3
Eugenol	502.0	502.0	502.0	1004.0	1004.0
Thymol	198.4	198.4	198.4	198.4	198.4
Oregano	218.6	232.2	232.2	218.6	232.2
Mugwort	>904.6	>904.6	>904.6	>904.6	>904.6
Geraniol	869.4	869.4	869.4	>869.4	>869.4
Linalool	>860.5	>860.5	>860.5	>860.5	>860.5
Citral	878.3	878.3	878.3	>878.3	>878.3
Curcumin	—	—	—	—	—

In addition, the MIC values of mugwort and linalool against five bacterial strains were more than 904.6 and 860.5 μg/mL, respectively, while the MIC values of geraniol and citral were 869.4 and 878.3 μg/mL, respectively, against *S. pullorum* and *E. coli*. For *K. pneumoniae*, the required concentrations of geraniol and citral were more than 869.4 and 878.3 μg/mL, respectively. Due to the color and solubility characteristics of curcumin causing too much deviation in the results, no further research was conducted using this compound.

Overall, the order of antimicrobial efficacy of the NAM employed in our experiments was established, as follows: thymol > oregano > carvacrol > trans-cinnamaldehyde > eugenol > geraniol > citral > linalool > mugwort.

### Effect of Natural Antimicrobials Alone and in Combination on the Growth Inhibition Rate of Poultry-Borne Pathogenic Bacteria

**FIGURE 1:**
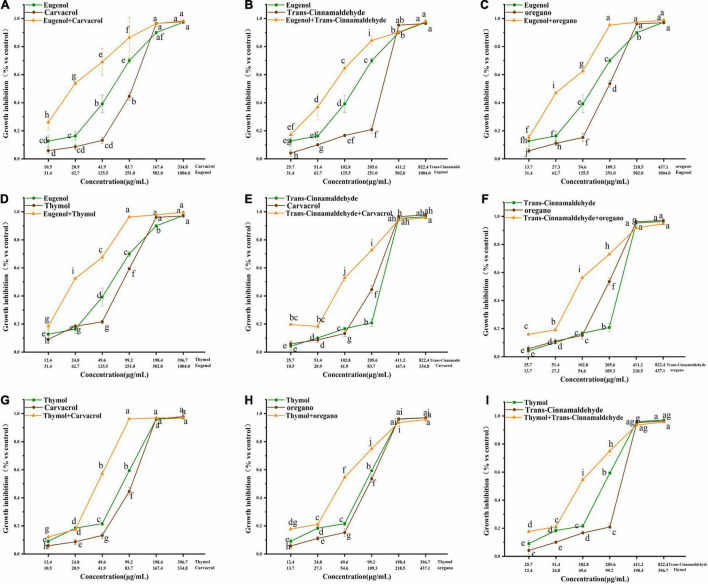
**(A–I)** Effect of single and combined of natural antimicrobials on the growth inhibition rate of *Salmonella pullorum* BNCC 19945. The significant differences among different concentrations or individual and combinations were analyzed using one-way ANOVA, followed by Duncan multiple comparisons (*P* < 0.05 represent significant differences). The differences between various concentrations and groups are marked by letters: the same letter represent no differences, while different letter indicate significant differences.

**FIGURE 2:**
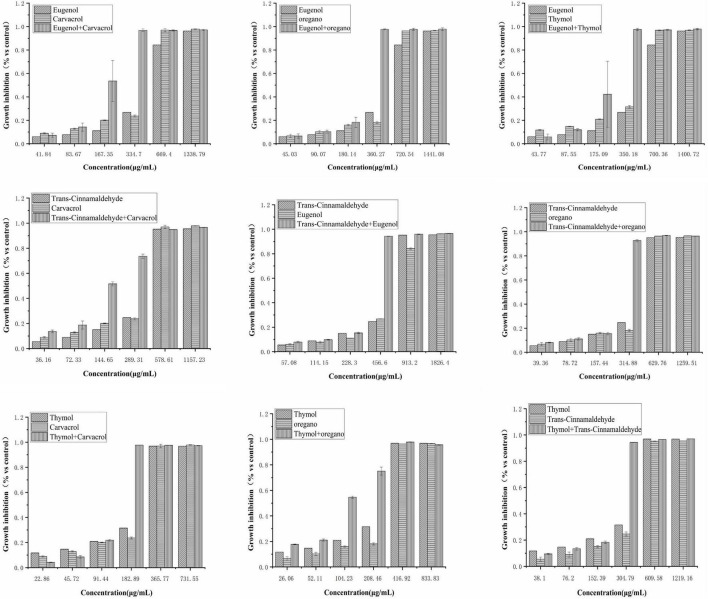
Effect of single and combined of natural antimicrobials on the growth inhibition rate of *Klebsiella pneumoniae* BNCC 102997.

**FIGURE 3:**
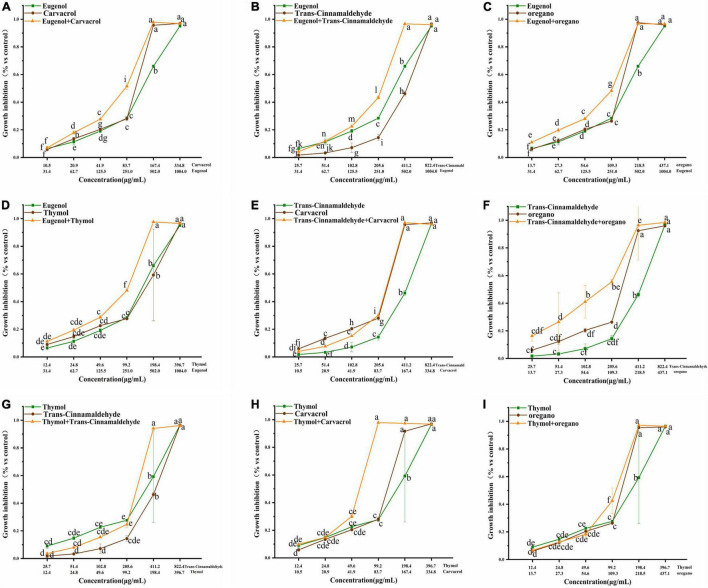
**(A–I)** Effect of single and combined of natural antimicrobials on the growth inhibition rate of Klebsiella pneumoniae sample 208G28. Note: The significant differences among different concentrations or individual and combinations were analyzed using one-way ANOVA, followed by Duncan multiple comparisons (*P* < 0.05 represents significant differences). The differences between various concentrations and groups are marked by letters: the same letters represent no differences, while different letters indicate significant differences.

The growth inhibitory effects of trans-cinnamaldehyde, eugenol, carvacrol, thymol, and oregano, used alone or in combination, were tested against poultry-borne pathogenic bacteria. Here, *S. pullorum* is presented as an example, and results related to other poultry-borne pathogenic bacteria are shown in Figures 1–3 (Supplementary Figures 1, 2 in Supplementary Information) Dose-response, effects of trans-cinnamaldehyde, eugenol, carvacrol, thymol, and oregano, individually and in combination with other NAM, were tested against *S. pullorum* (Figure 1I). The ANOVA data embodied the correlation between single components and the synergistic group. All tested NAM elicited a dose-dependent response from *S. pullorum*, indicating that higher tested doses resulted in greater inhibition. For example, when combining thymol and trans-cinnamaldehyde, the natural antibacterial effect of thymol increased along with the dose. The inhibition rate increased significantly (*p* < 0.05) at concentrations between 12.4 and 198.4 μg/mL, but no significant (*p* > 0.05) change was observed between 24.8 and 49.6 μg/mL. Beyond 49.6 μg/mL, the growth inhibition rate of *S. pullorum* increased significantly (*p* < 0.05), but no marked elevations were seen at the highest concentrations. Thus, despite continued increases in the concentration, the inhibition rate did not notably change, perhaps due to drug accumulation having reached saturation of solubility. Significant differences were observed between all five tested concentrations of trans-cinnamaldehyde. A combination of thymol and trans-cinnamaldehyde induced no significant increase in growth inhibition rate at the two lower concentrations; however, marked elevation were observed at the four higher concentrations.

### Evaluation of Combined Effects of Binary Drug Combinations Based on the Combination Index Model

According to the CI model, the relationship between the effects of drug combinations on the fraction affected (fa) levels of poultry-borne pathogens, which are generated using CompuSyn, and the CI index, was evaluated. Furthermore, the type and degree of interaction between the combined drugs (synergistic, antagonistic, or additive) were evaluated. Concentrations used in the individual natural antimicrobial exposures and the parameters of the curve fit of the concentration–response data are presented in Supplementary InformationSupplementary Table 1. The degree of interaction between drug combinations was evaluated according to the CI index, outcomes of which are presented in Table 2.

**TABLE 2:** Dose-effect relationship parameters and mean combination index (CI) values of drug combinations for bacterial strains.

Bacterial strains and drug combinations	Dose-effect parameters			Total dose	CI values		
			
	Dm	m	R	Range	IC10	IC50	IC90
***Salmonella pullorum* (BNCC 19945)**							
Eugenol + Carvacrol	86.2506	1.59023 ± 0.09411	0.99307	13.5404–767.517	0.67491	0.81650	0.99792
Eugenol + Trans-Cinnamaldehyde	155.894	1.43973 ± 0.03833	0.99859	20.1670–1743.38	0.77317	1.03175	1.37994
Eugenol + Oregano	100.444	1.81494 ± 0.20360	0.97574	19.8313–681.892	0.84924	0.86637	0.89352
Eugenol + Thymol	84.2626	1.83043 ± 0.24564	0.96582	16.8665–562.845	0.87214	0.82863	0.78784
Trans-Cinnamaldehyde + Carvacrol	136.531	1.53598 ± 0.19330	0.96975	20.0770–1312.46	0.93327	1.26604	1.72237
Trans-Cinnamaldehyde + Oregano	147.997	1.43921 ± 0.11949	0.98649	19.1312–1656.53	0.82314	1.23226	1.85072
Thymol + Carvacrol	68.8409	1.86595 ± 0.33506	0.94115	14.2081–443.505	1.14654	1.16855	1.19772
Thymol + Trans-Cinnamaldehyde	133.841	1.49172 ± 0.13957	0.98294	18.5932–1375.97	0.99442	1.29890	1.69742
Thymol + Oregano	64.6638	1.57822 ± 0.30114	0.93428	10.0094–585.081	0.74637	0.94409	1.20156
***Klebsiella pneumoniae* (BNCC 102997)**							
Eugenol + Carvacrol	138.790	2.09303 ± 0.46034	0.91536	33.9939–730.520	0.99014	0.88437	0.79496
Eugenol + Oregano	177.707	2.22753 ± 0.52506	0.90452	47.3844–846.112	1.14294	0.98976	0.86954
Eugenol + Thymol	161.538	2.25024 ± 0.45985	0.92567	43.6516–757.113	1.23887	1.04267	0.88445
Trans-Cinnamaldehyde + Carvacrol	135.255	1.50114 ± 0.25262	0.94776	20.0770–1312.46	0.67544	1.04261	1.61535
Trans-Cinnamaldehyde + Eugenol	250.082	2.04069 ± 0.42833	0.92205	59.0820–1373.59	1.15615	1.11964	1.10599
Trans-Cinnamaldehyde + oregano	171.750	2.03354 ± 0.41458	0.92598	40.3706–949.011	1.04338	1.14762	1.26283
Thymol + Carvacrol	108.009	2.42118 ± 0.66116	0.87764	32.0117–453.920	1.71083	1.44465	1.21998
Thymol + Oregano	121.354	2.20145 ± 0.62206	0.87059	41.3595–543.334	1.40981	1.35061	1.29510
Thymol + Tri-Cinnamaldehyde	151.900	1.97656 ± 0.42493	0.91868	34.2451–881.724	1.07444	1.17502	1.28844
***Klebsiella pneumoniae* (Sample 208G28)**							
Eugenol + Carvacrol	201.467	1.89262 ± 0.28499	0.95752	42.5160–1264.31	1.58686	1.45944	1.37216
Eugenol + Trans-Cinnamaldehyde	327.646	1.99558 ± 0.30031	0.95757	74.9227–1870.25	1.48377	1.21874	1.02321
Eugenol + Oregano	207.220	1.71898 ± 0.31420	0.93921	37.3704–1565.51	1.24703	1.10779	0.98418
Eugenol + Thymol	168.003	1.84977 ± 0.28921	0.95443	34.1991–1100.14	1.31347	1.00038	0.76406
Trans-Cinnamaldehyde + Carvacrol	241.251	2.08475 ± 0.42720	0.92530	58.7605–1278.23	1.68797	1.28334	1.00857
Trans-Cinnamaldehyde + Thymol	262.282	2.07583 ± 0.40299	0.93220	63.4964–1399.65	2.01296	1.47117	1.11865
Trans-Cinnamaldehyde + Oregano	151.449	1.22877 ± 0.36379	0.86047	13.7907–2563.63	0.44616	0.74571	1.27126
Thymol + Carvacrol	81.8056	2.04616 ± 0.51423	0.89349	19.4013–447.280	1.36650	1.08952	0.90436
Thymol + Oregano	144.535	2.01481 ± 0.34051	0.94735	33.5195–811.429	2.06599	1.67412	1.41303
***Escherichia coli* (BNCC 336435)**							
Eugenol + Carvacrol	193.829	2.12197 ± 0.37814	0.94196	48.3939–997.372	1.33135	1.11517	0.93778
Eugenol + Trans-Cinnamaldehyde	315.754	1.99181 ± 0.27167	0.96475	72.0019–1808.32	1.28793	1.38033	1.49217
Eugenol + Oregano	214.299	2.04562 ± 0.34978	0.94620	50.8045–1172.22	1.23285	1.12353	1.02453
Trans-Cinnamaldehyde + Carvacrol	247.809	2.12199 ± 0.35132	0.94931	61.8724–1275.11	2.08756	1.92665	1.82263
Trans-Cinnamaldehyde + Thymol	203.414	1.88765 ± 0.26231	0.96348	42.7513–1282.72	1.37847	1.58517	1.83170
Trans-Cinnamaldehyde + Oregano	246.195	1.70246 ± 0.40870	0.90148	43.6674–1896.82	1.38875	1.72987	2.18541
Thymol + Carvacrol	98.7540	2.45022 ± 0.39336	0.95213	29.6935–408.022	1.64690	1.24451	0.94771
Thymol + Eugenol	137.803	1.67057 ± 0.20677	0.97070	23.6481–1103.91	0.65760	0.80731	0.99169
Thymol + Oregano	87.8112	1.81608 ± 0.34048	0.93634	17.3549–595.414	0.88818	0.95535	1.03011
***Escherichia coli* (Sample 8G4)**							
Eugenol + Carvacrol	159.513	1.84864 ± 0.38423	0.92340	32.4390–1045.74	1.18294	1.20699	1.23154
Eugenol + Trans-Cinnamaldehyde	364.665	2.00579 ± 0.24204	0.97209	84.0165–2063.19	2.56456	1.90057	1.47564
Eugenol + Oregano	167.008	1.67010 ± 0.33875	0.92666	28.6459–1338.64	0.95955	0.99297	1.04400
Trans-Cinnamaldehyde + Carvacrol	209.647	1.73728 ± 0.25734	0.95881	38.4966–1550.47	1.96451	1.72447	1.58592
Trans-Cinnamaldehyde + Thymol	251.378	2.00142 ± 0.23853	0.97275	57.7305–1427.63	2.35732	1.78308	1.44882
Trans-Cinnamaldehyde + Oregano	228.159	1.51952 ± 0.32655	0.91874	32.8616–2247.71	1.56494	1.44135	1.33787
Thymol + Carvacrol	105.968	2.18673 ± 0.41177	0.93583	27.5675–519.450	1.50877	1.34124	1.19554
Thymol + Eugenol	182.791	1.71311 ± 0.18453	0.97757	30.4115–1123.35	1.02955	1.20831	1.42193
Thymol + Oregano	141.769	2.36400 ± 0.45496	0.93325	40.7994–616.850	1.79340	1.30866	0.98613

**FIGURE 4:**
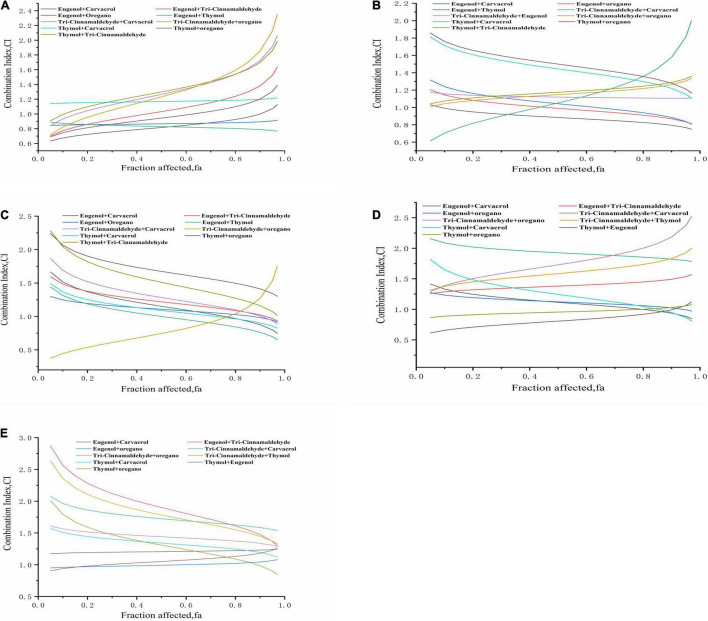
Evaluation of combined effects of binary natural antimicrobials combinations based on CI model. Combination index plot (fa–CI plot) for pairwise drug combinations: **(A)** *Salmonella pullorum* BNCC 19945; **(B)** *Klebsiella pneumoniae* BNCC 102997; **(C)** *K. pneumoniae* Sample 208G28; **(D)** *Escherichia coli* BNCC 336435; **(E)** *E. coli* Sample 8G4.

Synergistic interaction was examined by calculating the CI values of the NAM against poultry-borne pathogenic bacteria evaluated in this study; values are shown in Figures 4A–E, revealing the degree of synergistic effect. *S. pullorum* (Figure 4A) is presented as an example. Following exposure to the combination of eugenol and thymol (or oregano) for 24 h, a stable synergistic effect was observed between 0.05 and 0.97 fa levels. A combination of thymol and carvacrol displayed a slight antagonistic behavior between 0.05 and 0.97 fa levels. A combination of eugenol and carvacrol showed very strong synergism at the 0.05 fa level; an additive property was seen at fa levels between 0.75 and 0.97, which became synergistic with increasing degree at lower fa levels. The combination of eugenol and trans-cinnamaldehyde exhibited a very strong synergistic effect, with an fa level of 0.05, which changed to a strong synergistic effect, with the fa level rising to 0.25. An additive effect was perceived between 0.30 and 0.65 fa levels, and higher fa levels yielded antagonistic effects, which became slightly antagonistic at fa levels between 0.70 and 0.80 and moderately antagonistic from 0.85 to 0.97. The combination of trans-cinnamaldehyde and carvacrol showed very potent synergism at an fa level of 0.05, an additive effect at fa levels from 0.10 to 0.30, and an antagonistic effect between 0.35 and 0.97. In the trans-cinnamaldehyde and oregano mixture, the 0.05, 0.10, and 0.15 fa levels gave strong synergistic effects, with an additive effect observed at fa levels from 0.20 to 0.40, and higher fa levels (between 0.45 and 0.97) yielding antagonistic effects. Combined thymol and trans-cinnamaldehyde showed an additive effect between 0.05 and 0.25 fa levels, and an antagonistic effect from 0.30 to 0.97. Lastly, the combination of thymol and oregano exhibited a strong synergism between 0.05 and 0.40 fa levels, changing to an additive property with the fa level rising to 0.85; fa levels at 0.90, 0.95, and 0.97 gave antagonistic effects.

Based on the antibacterial effects of these combination groups, we derived several consistent conclusions. First, eugenol showed a more potent synergistic property than the other NAM, although the antibacterial property of eugenol alone was the weakest among all five compounds. The synergistic outcomes of trans-cinnamaldehyde and eugenol may be due to the action of eugenol/trans-cinnamaldehyde on enzymes or diverse proteins. Moreover, the synergistic properties of carvacrol/eugenol and thymol/eugenol may be due to thymol and carvacrol disintegrating the outer membrane of *S. pullorum*, enabling eugenol entry into the cytoplasm to interact with proteins. Second, thymol showed a potent antibacterial behavior against poultry-borne pathogens, while its interactive effects with other compounds were not significant. Conversely, the paired combination of carvacrol and thymol (total dose 183 μg/mL) showed synergistic effects against *E. coli*, *S. pullorum*, and *K. pneumoniae*. Additionally, for *S. pullorum*, all CI values were less than 1.0, which indicates zero antagonism between eugenol and thymol (or oregano). Given these results, the paired combinations at certain concentrations present synergistic AMA, at least against majority of the test bacteria.

## Discussion

In this study, we aimed to identify several synergistic NAM combinations that can inhibit the growth of poultry-borne bacterial pathogens. Eight NAM, often referred to as active aromatic compounds, can inhibit bacterial growth; these include carvacrol (Pei et al., 2009; Palaniappan and Holley, 2010; Pinheiro et al., 2017; Walsh et al., 2019), trans-cinnamaldehyde (Kollanoor Johny et al., 2010; Palaniappan and Holley, 2010; Hermans et al., 2011), thymol (Oussalah et al., 2007), oregano (Friedman et al., 2002; Aslim and Yucel, 2008; Ravishankar et al., 2008), citral (Tang et al., 2018), curcumin (Ravishankar et al., 2008; Navarro et al., 2015), eugenol (Charan Raja, 2015; Liu et al., 2015), and geraniol (Tang et al., 2018). However, data related to the associations between different NAM are limited, and an extensive investigation is needed to provide empirical data on the bactericidal effects of combinational therapies.

Here, five NAM (trans-cinnamaldehyde, eugenol, carvacrol, thymol, and oregano) were found active against tested strains of poultry-borne bacteria *in vitro*. We noted that thymol was more effective than the other four compounds, exhibiting the lowest MIC value (198 μg/mL) against five bacterial strains. Pei et al. (2009) used carvacrol, cinnamaldehyde, eugenol, and thymol against *E. coli* and observed MIC values of 9.7, 2.6, 3.0, and 2.6 mM, respectively. In the present study, the MIC values of eugenol and trans-cinnamaldehyde were comparable to those of Pei et al. (2009). Our results further agreed with those of Olasupo et al. (2003), wherein the MIC values of 1.5 and 1.0 mM, 2.5 and 3.0 mM, and 1.2 and 1.0 mM for carvacrol, eugenol, and thymol, respectively, inhibited *E. coli* and *Salmonella typhimurium*. Our data also correlates with those by Rivas et al. (2010), who demonstrated the AMA of thymol and carvacrol against verocytotoxigenic *E. coli* and *E. coli* O157:H7 at 500 μg/mL, as well as those of Helander et al. (1998) showing that thymol, cinnamaldehyde, and carvacrol inhibited *E. coli* and *S. typhimurium* at concentrations ranging from 1 to 3 mM. Miladi et al. (2017) used thymol and carvacrol against a panel of clinical and foodborne pathogens and observed MIC values ranging from 32–128 mg/L and 32–64 mg/L, respectively. However, the MICs of thymol and carvacrol in our study were slightly higher for gram-negative bacteria. The discrepancy in AMA could be attributed to testing the compounds against strains with elevated resistance to the examined antibiotics. Additionally, the mechanism of antibacterial action may vary between different bacteria (Pei et al., 2009). Oussalah et al. (2007) studied the AMA of 28 essential oils against four pathogens and showed that the majority of oils inhibited *E. coli*, *S. typhimurium*, *Listeria monocytogenes*, and *Staphylococcus aureus*. Among the bacteria tested herein, *S. pullorum* was most responsive to the employed NAM, and the concentrations of trans-cinnamaldehyde and carvacrol required for inhibition were less than for *E. coli* and *K. pneumoniae*. In addition, previous reports showed that NAM also demonstrated important antifungal (*Aspergillus flavus*) properties (López-Malo et al., 2002; Lopez-Malo et al., 2005, 2006; Garcia-Garcia et al., 2011). A. flavus exhibited higher sensitivity to thymol, eugenol, carvacrol, sodium bisulfite, and sodium benzoate (at pH 3.5) than to vanillin or citral. Moreover, liquid model systems based on the binary and ternary mixtures on *Listeria innocua* inactivation were used to evaluate bactericidal effect of three natural agents (carvacrol, thymol, and eugenol). Results showed that the most effective individual antimicrobial agent was carvacrol, followed by thymol and eugenol, and the most effective binary mixture was 75 mg/kg of carvacrol mixed with 62.5 mg/kg of thymol.

The AMA of thymol, oregano, trans-cinnamaldehyde, eugenol, and carvacrol exhibited comparable results, although our results concluded that the order of AMA efficacy was thymol > oregano > carvacrol > trans-cinnamaldehyde > eugenol. However, Michiels et al. (2007) reported that the AMA efficacy order against *E. coli* in small intestinal simulations was cinnamaldehyde > carvacrol > thymol, while Friedman et al. (2002) observed the order carvacrol > cinnamaldehyde > thymol against *E. coli* and *Salmonella enterica*, using a microplate assay. Olasupo et al. (2003) found that thymol was more effective than other essential oils, and the AMA order of natural organic compounds against *E. coli* and *S. typhimurium* was carvacrol > eugenol > cinnamic acid > diacetyl, evaluated using a checkerboard assay and the Bioscreen C instrument. These differences in AMA order observed in various studies may be attributed to the various evaluation techniques performed to assess the AMA. Moreover, several factors such as culture medium from different manufacturers, growth curve discrepancies, differences in bacterial growth phases among the different bacteria, pH, incubation temperature and time, and volume of the inoculum could affect experimental outcomes.

Previous studies have revealed the different techniques used to evaluate the synergistic behavior of NAM. Many researchers have used the checkerboard technique based on the FIC and EC indices to evaluate combined effects (Si et al., 2008). Our research used the broth microdilution assay associated with the checkerboard method based on the CI and fa indices to evaluate combined effects (Chou, 2006). Synergy is defined as the synergistic effect of two compounds that is larger than the sum of the effects of each individual compound (Kobilinsky et al., 2007). The efficacy of synergistic associations of plant-based antimicrobials are well-established (Brenes and Roura, 2010) and is based on the principle that combining active compounds augments AMA, while diminishing toxicity and amount of substance in the final formula. Pei et al. (2009) defined the synergistic effects of thymol/carvacrol, cinnamaldehyde/eugenol, thymol/eugenol, and carvacrol/eugenol. Likewise, the effective concentrations of the five components evaluated in this study could be significantly lower in combination than when used alone.

Little is known about the exact mechanism by which NAM reduce antibiotic resistance; however, it is likely due to structural alterations occurring in the resistant bacteria after antimicrobial exposure. Recently, Nowotarska et al. (2014) used model membranes to demonstrate penetration of the phenolic group of carvacrol into the lipid molecules of the bacterial membrane. Similarly, Stratakos et al. (2018) provided antimicrobial mechanistic insights on carvacrol, demonstrating its significant efficacy against clinically relevant Shiga toxin-synthesizing *E. coli* (STEC) and non-STEC strains. Gayan et al. (2020) examined the synergistic effect of carvacrol in combination with mild heat and showed pronounced inactivation of the heat-resistant *E. coli* ATCC 43888. Lambert et al. (2001) found that thymol and carvacrol are hydrophobic and susceptible to disturbing the outer membrane of gram-negative bacteria, enhancing permeability of the cytoplasmic membrane to adenosine triphosphate, releasing lipopolysaccharides, and abrogating the inhibitory effects of protective enzymes. Accordingly, several studies have reported that the carbonyl group on trans-cinnamaldehyde might bind to proteins, preventing amino acid decarboxylase activities, while the hydroxyl (-OH) group on eugenol may bind to proteins, causing structural breakdown, deactivating enzyme, ultimately stimulating cell membrane lysis and bacterial death (Hemaiswarya et al., 2008; Palaniappan and Holley, 2010; Quinto et al., 2019). We hypothesized that the mechanisms of AMA may be different, or that the combinational action may alter against various bacteria. Additional investigations are warranted to enhance the understanding of the mechanism behind the synergistic properties of NAM.

Several previous reports have indicated that NAM can protect against bacterial infection and lower pathogen virulence *in vitro*. However, few studies have demonstrated their effectiveness in living animal models (Abudabos et al., 2019; Liu et al., 2020). Previous studies have demonstrated the positive effects of various phytogenic feed additives in broilers (Si et al., 2006; Sengül et al., 2008; Alzawqari et al., 2016; Khan et al., 2019). Production parameters improved in birds supplemented with herbal oil, which consists of different important alkaloids (Alzawqari et al., 2016; Khan et al., 2019). The results of the present study are similar those of Sengül et al. (2008), who showed that the growth performance of Japanese quail was not negatively affected by supplementation with thyme extract. Rather, these parameters improved notably with addition of thyme extract products to their diets. In addition, Si et al. (2006) used thymol, carvacrol, and trans-cinnamaldehyde against *Salmonella* in pigs and found that higher concentrations were required to maintain AMA when introduced in the diet.

In summary, we highlighted the potential use of thymol and carvacrol as combined treatment for controlling poultry-borne bacterial pathogens. We further concluded that the antibacterial abilities of NAM were enhanced when used in combination, and the minimum effective concentrations could be decreased substantially. In our study, we provided empirical and comprehensive data for synergistic outcomes. Further studies should establish the mode of action of combined treatments and assess the effectiveness of these combined compounds in animal intestinal models and animals.

## Conclusion

The activity of NAM alone and in combination against common poultry pathogens (*S. pullorum*, *E. coli*, and *K. pneumoniae*) was evaluated using broth dilution and checkerboard methods, and the types of interactions between two combined drugs were evaluated based on CI model. The comprehensive analysis showed that the order of the inhibitory effects of the NAM on pathogens was: thymol > oregano > carvacrol > trans-cinnamaldehyde > eugenol > geraniol > citral > linalool > mugwort. Thymol had the strongest inhibitory effect on common pathogens and showed a good antibacterial effect. The combined interaction of NAM (carvacrol, eugenol, trans-cinnamaldehyde, oregano, and thymol) was evaluated using the CI model, and the synergistic effect of the combination of carvacrol and thymol was observed to have better inhibition effect on common pathogens *in vitro*. Thus, the combination of thymol and carvacrol was selected for comprehensive analysis to study the effect of NAM on microbial resistance in broilers.

## Data Availability Statement

The original contributions presented in the study are included in the article/Supplementary Material, further inquiries can be directed to the corresponding author.

## Author Contributions

SY conceived and designed the experiments. RZ and RL performed statistical and data analysis. QL and JW contributed to the reagents and materials. XL wrote this manuscript and prepared the original draft. YC and YW provided the detection samples in this research. All authors have read and agreed to the published version of the manuscript.

## Conflict of Interest

The authors declare that the research was conducted in the absence of any commercial or financial relationships that could be construed as a potential conflict of interest.

## Publisher’s Note

All claims expressed in this article are solely those of the authors and do not necessarily represent those of their affiliated organizations, or those of the publisher, the editors and the reviewers. Any product that may be evaluated in this article, or claim that may be made by its manufacturer, is not guaranteed or endorsed by the publisher.

## Abbreviations

CI: combination index
MIC: minimum inhibitory concentrations
CLSI: Clinical and Laboratory Standards Institute
EC: effect of the combination.
