# Supplementary material for: Synergistic Interaction Between Paired Combinations of Natural Antimicrobials Against Poultry-Borne Pathogens
Source: Front Microbiol. 2022 May 4;13:811784. doi: 10.3389/fmicb.2022.811784 (PMC9115557; doi:10.3389/fmicb.2022.811784)
Supplement: Supplementary file 1 [file Data_Sheet_1.docx]

**Figure Captions:**

Fig.S1 Effect of single and combined of natural antimicrobials on the growth inhibition rate of Poultry-borne bacteria pathogens(*Escherichia coli*（BNCC 336435）)

Fig.S2 Effect of single and combined of natural antimicrobials on the growth inhibition rate of Poultry-borne bacteria pathogens (*Escherichia coli*（sample 8G4）)

**Figure. S1**


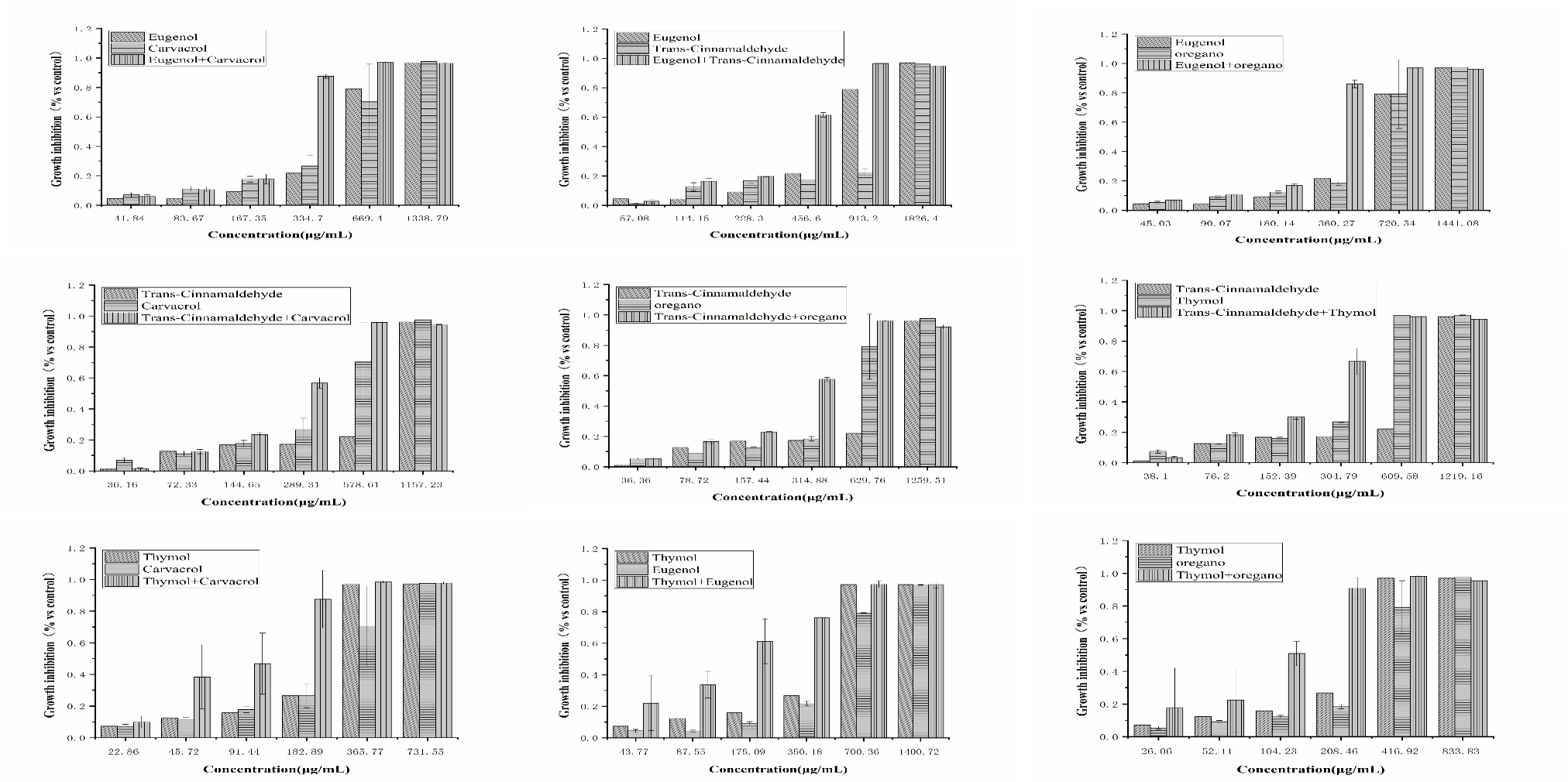
**Figure. S1 Effect of single and combined of natural antimicrobials on the growth inhibition rate of *Escherichia coli* (BNCC 336435）)**

**Figure. S2**

**Figure. S2 Effect of single and combined of natural antimicrobials on the growth inhibition rate of *Escherichia coli* (sample 8G4)**

**
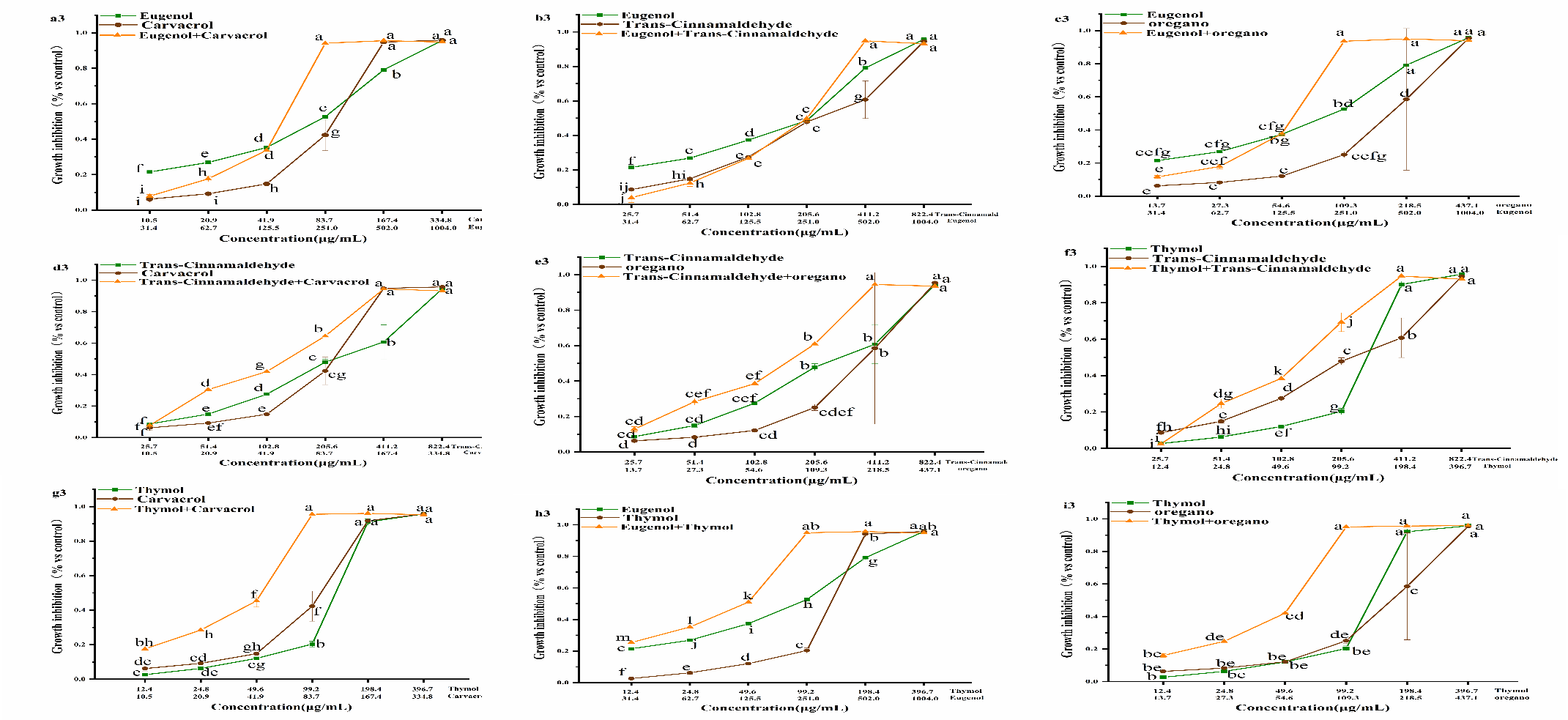
**

**Table SI1** Concentrations used in single-natural antimicrobial exposures and the parameters of the curve fit of the concentration–response data for each individual natural antimicrobial.

| Antimicrobials | Concentration range (mg/mL) | IC50 (μg/mL) | R^2^ |
| --- | --- | --- | --- |
| Carvacrol | 0.01046 – 1.91373 | 83.7026 | 0.98976 |
| Trans-Cinnamaldehyde | 0.02570 – 2.0751 | 205.6043 | 0.99531 |
| Eugenol | 0.03137 – 2.09216 | 250.9961 | 0.96403 |
| Thymol | 0.01240 – 2.0451 | 99.1847 | 0.94006 |
| oregano | 0.01366 – 1.85573 | 109.2741 | 0.99897 |

**Table. S****I2** Impact of solo and paired combinations of natural antimicrobials on the growth inhibition rate of *Salmonella pullorum*（BNCC 19945）

|  | 1 | 2 | 3 | 4 | 5 | 6 |
| --- | --- | --- | --- | --- | --- | --- |
| **Carvacrol** | 10.46282 | 20.92564 | 41.85129 | 83.70257 | 167.4051 | 334.8103 |
| **CAR** | 0.072986 | 0.097616 | 0.122669 | 0.435689 | 0.961123 | 0.977853 |
| **AVER** | 0.058795 | 0.086632 | 0.132637 | 0.445535 | 0.965176 | 0.978229 |
| **SD** | 0.020969 | 0.019235 | 0.021605 | 0.025849 | 0.001962 | 0.00013 |
|  |  |  |  |  |  |  |
|  | 1 | 2 | 3 | 4 | 5 | 6 |
| **Eugenol** | 31.37452 | 62.74903 | 125.4981 | 250.9961 | 501.9923 | 1003.985 |
| **EUG** | 0.121494 | 0.128434 | 0.355857 | 0.71183 | 0.900836 | 0.973343 |
| **AVER** | 0.127611 | 0.163337 | 0.39228 | 0.699731 | 0.89951 | 0.973421 |
| **SD** | 0.036854 | 0.030686 | 0.063297 | 0.017726 | 0.002415 | 0.000242 |
|  |  |  |  |  |  |  |
|  | 1 | 2 | 3 | 4 | 5 | 6 |
| **trans-cinnamaldehyde** | 25.70054 | 51.40108 | 102.8022 | 205.6043 | 411.2086 | 822.4172 |
| **TC** | 0.067082 | 0.113584 | 0.169774 | 0.224202 | 0.952676 | 0.964272 |
| **AVER** | 0.041844 | 0.100613 | 0.167171 | 0.208897 | 0.952873 | 0.964691 |
| **SD** | 0.027056 | 0.007653 | 0.006496 | 0.026117 | 0.001152 | 0.000235 |
|  |  |  |  |  |  |  |
|  | 1 | 2 | 3 | 4 | 5 | 6 |
| **Oregano** | 13.65926 | 27.31852 | 54.63704 | 109.2741 | 218.5482 | 437.0963 |
| **ORI** | 0.049695 | 0.097609 | 0.13802 | 0.461715 | 0.957232 | 0.970999 |
| **AVER** | 0.057216 | 0.110868 | 0.152832 | 0.535794 | 0.96229 | 0.970568 |
| **SD** | 0.016329 | 0.016438 | 0.02894 | 0.025774 | 0.000718 | 0.000805 |
|  |  |  |  |  |  |  |
|  | 1 | 2 | 3 | 4 | 5 | 6 |
| **Thymol** | 12.39808 | 24.79616 | 49.59233 | 99.18465 | 198.3693 | 396.7386 |
| **THY** | 0.107629 | 0.179354 | 0.202557 | 0.58356 | 0.957746 | 0.969677 |
| **AVER** | 0.090786 | 0.184302 | 0.216192 | 0.593778 | 0.959927 | 0.970337 |
| **SD** | 0.022939 | 0.011853 | 0.01268 | 0.007067 | 0.000553 | 0.0009 |
|  |  |  |  |  |  |  |
|  | 1 | 2 | 3 | 4 | 5 | 6 |
| **Eugenol** | 31.37452 | 62.74903 | 125.4981 | 250.9961 | 501.9923 | 1003.985 |
| **carvacrol** | 10.46282 | 20.92564 | 41.85129 | 83.70257 | 167.4051 | 334.8103 |
|  | 41.83734 | 83.67468 | 167.3494 | 334.6987 | 669.3974 | 1338.795 |
| **AVER** | 0.260562 | 0.539745 | 0.691276 | 0.864382 | 0.964507 | 0.982474 |
| **SD** | 0.054938 | 0.013107 | 0.095924 | 0.163449 | 0.023761 | 0.002444 |
|  |  |  |  |  |  |  |
|  | 1 | 2 | 3 | 4 | 5 | 6 |
| **Eugenol** | 31.37452 | 62.74903 | 125.4981 | 250.9961 | 501.9923 | 1003.985 |
| **TC** | 25.70054 | 51.40108 | 102.8022 | 205.6043 | 411.2086 | 822.4172 |
|  | 57.07506 | 114.1501 | 228.3002 | 456.6004 | 913.2009 | 1826.402 |
| **AVER** | 0.173406 | 0.369723 | 0.646385 | 0.843049 | 0.902776 | 0.978769 |
| **SD** | 0.024928 | 0.092705 | 0.036652 | 0.028578 | 0.012889 | 0.007604 |
|  |  |  |  |  |  |  |
|  | 1 | 2 | 3 | 4 | 5 | 6 |
| **Eugenol** | 31.37452 | 62.74903 | 125.4981 | 250.9961 | 501.9923 | 1003.985 |
| **Oregano** | 13.65926 | 27.31852 | 54.63704 | 109.2741 | 218.5482 | 437.0963 |
|  | 45.03378 | 90.06755 | 180.1351 | 360.2702 | 720.5404 | 1441.081 |
| **AVER** | 0.151792 | 0.470538 | 0.625999 | 0.953586 | 0.97393 | 0.988528 |
| **SD** | 0.015634 | 0.005407 | 0.019049 | 0.002776 | 0.000654 | 0.004146 |
|  |  |  |  |  |  |  |
|  | 1 | 2 | 3 | 4 | 5 | 6 |
| **Eugenol** | 31.37452 | 62.74903 | 125.4981 | 250.9961 | 501.9923 | 1003.985 |
| **Thymol** | 12.39808 | 24.79616 | 49.59233 | 99.18465 | 198.3693 | 396.7386 |
|  | 43.7726 | 87.5452 | 175.0904 | 350.1808 | 700.3616 | 1400.723 |
| **AVER** | 0.184551 | 0.524162 | 0.675848 | 0.962784 | 0.978443 | 0.996088 |
| **SD** | 0.020858 | 0.006652 | 0.027228 | 0.004746 | 0.002509 | 0.002037 |
|  |  |  |  |  |  |  |
|  | 1 | 2 | 3 | 4 | 5 | 6 |
| **TC** | 25.70054 | 51.40108 | 102.8022 | 205.6043 | 411.2086 | 822.4172 |
| **carvacrol** | 10.46282 | 20.92564 | 41.85129 | 83.70257 | 167.4051 | 334.8103 |
|  | 36.16336 | 72.32672 | 144.6534 | 289.3069 | 578.6138 | 1157.228 |
| **AVER** | 0.196545 | 0.183059 | 0.529303 | 0.726761 | 0.941235 | 0.957022 |
| **SD** | 0.00491 | 0.016871 | 0.022568 | 0.003937 | 0.000537 | 0.001131 |
|  |  |  |  |  |  |  |
|  | 1 | 2 | 3 | 4 | 5 | 6 |
| **TC** | 25.70054 | 51.40108 | 102.8022 | 205.6043 | 411.2086 | 822.4172 |
| **Eugenol** | 31.37452 | 62.74903 | 125.4981 | 250.9961 | 501.9923 | 1003.985 |
|  | 57.07506 | 114.1501 | 228.3002 | 456.6004 | 913.2009 | 1826.402 |
| **AVER** | 0.13155 | 0.213187 | 0.567156 | 0.735874 | 0.930516 | 0.952967 |
| **SD** | 0.019916 | 0.007786 | 0.010732 | 0.003115 | 0.000205 | 0.000342 |
|  |  |  |  |  |  |  |
|  | 1 | 2 | 3 | 4 | 5 | 6 |
| **TC** | 25.70054 | 51.40108 | 102.8022 | 205.6043 | 411.2086 | 822.4172 |
| **Oregano** | 13.65926 | 27.31852 | 54.63704 | 109.2741 | 218.5482 | 437.0963 |
|  | 39.3598 | 78.7196 | 157.4392 | 314.8784 | 629.7568 | 1259.514 |
| **AVER** | 0.160175 | 0.191359 | 0.563799 | 0.730852 | 0.917961 | 0.947291 |
| **SD** | 0.004575 | 0.010916 | 0.008366 | 0.005719 | 0.001544 | 0.000177 |
|  |  |  |  |  |  |  |
|  | 1 | 2 | 3 | 4 | 5 | 6 |
| **TC** | 25.70054 | 51.40108 | 102.8022 | 205.6043 | 411.2086 | 822.4172 |
| **Thymol** | 12.39808 | 24.79616 | 49.59233 | 99.18465 | 198.3693 | 396.7386 |
|  | 38.09862 | 76.19724 | 152.3945 | 304.789 | 609.5779 | 1219.156 |
| **AVER** | 0.144198 | 0.209486 | 0.514234 | 0.712317 | 0.933523 | 0.951657 |
| **SD** | 0.006093 | 0.015473 | 0.015403 | 0.009443 | 0.000561 | 0.002426 |
|  |  |  |  |  |  |  |
|  | 1 | 2 | 3 | 4 | 5 | 6 |
| **Thymol** | 12.39808 | 24.79616 | 49.59233 | 99.18465 | 198.3693 | 396.7386 |
| **carvacrol** | 10.46282 | 20.92564 | 41.85129 | 83.70257 | 167.4051 | 334.8103 |
|  | 22.8609 | 45.72181 | 91.44361 | 182.8872 | 365.7744 | 731.5489 |
| **AVER** | 0.12167 | 0.174356 | 0.572159 | 0.962213 | 0.969203 | 0.97116 |
| **SD** | 0.001834 | 0.004122 | 0.01628 | 0.001087 | 0.000667 | 0.00066 |
|  |  |  |  |  |  |  |
|  | 1 | 2 | 3 | 4 | 5 | 6 |
| **Thymol** | 12.39808 | 24.79616 | 49.59233 | 99.18465 | 198.3693 | 396.7386 |
| **Eugenol** | 31.37452 | 62.74903 | 125.4981 | 250.9961 | 501.9923 | 1003.985 |
|  | 43.7726 | 87.5452 | 175.0904 | 350.1808 | 700.3616 | 1400.723 |
| **AVER** | 0.209042 | 0.250445 | 0.601587 | 0.943014 | 0.959365 | 0.964789 |
| **SD** | 0.003991 | 0.022527 | 0.001298 | 0.010627 | 0.000574 | 0.000421 |
|  |  |  |  |  |  |  |
| **EC** | 1 | 2 | 3 | 4 | 5 | 6 |
| **Thymol** | 12.39808 | 24.79616 | 49.59233 | 99.18465 | 198.3693 | 396.7386 |
| **TC** | 25.70054 | 51.40108 | 102.8022 | 205.6043 | 411.2086 | 822.4172 |
|  | 38.09862 | 76.19724 | 152.3945 | 304.789 | 609.5779 | 1219.156 |
| **AVER** | 0.177321 | 0.210992 | 0.546676 | 0.751178 | 0.936091 | 0.956978 |
| **SD** | 0.00297 | 0.008383 | 0.008421 | 0.031818 | 0.001658 | 0.002177 |
|  |  |  |  |  |  |  |
|  | 1 | 2 | 3 | 4 | 5 | 6 |
| **Thymol** | 12.39808 | 24.79616 | 49.59233 | 99.18465 | 198.3693 | 396.7386 |
| **Oregano** | 13.65926 | 27.31852 | 54.63704 | 109.2741 | 218.5482 | 437.0963 |
|  | 26.05734 | 52.11468 | 104.2294 | 208.4587 | 416.9175 | 833.8349 |
| **AVER** | 0.177321 | 0.210992 | 0.546676 | 0.751178 | 0.936091 | 0.956978 |
| **SD** | 0.00297 | 0.008383 | 0.008421 | 0.031818 | 0.001658 | 0.002177 |
